# Relationship between mental fatigue and burnout syndrome in remote workers during the COVID-19 pandemic: an integrative review

**DOI:** 10.47626/1679-4435-2022-1003

**Published:** 2023-11-24

**Authors:** Gabriela P. Urrejola-Contreras

**Affiliations:** Escuela de Ciencias de la Salud, Universidad Viña del Mar, Viña del Mar, Chile

**Keywords:** professional burnout, teleworking, occupational diseases, occupational health, esgotamento profissional, teletrabalho, doenças profissionais, saúde ocupacional

## Abstract

This study aimed to analyze the scientific evidence available in the literature addressing the relationship between mental fatigue and burnout associated with teleworking during the COVID-19 pandemic through an integrative review. This review searched the following databases: PubMed, Scopus, Taylor & Francis, Embase, ScienceDirect, and SciELO using the DeCS and MeSH health sciences descriptors. The included articles were published between March and December 2021, during the pandemic. Of a total of 224 results, 215 articles were excluded and 9 were considered for the preparation of this integrative review. Mental fatigue was related to technostress, somatic symptoms such as anxiety and insomnia (p < 0.05), and loss of motivation (p < 0.05). Burnout was positively associated with work overload, high interdependence, and lower role clarity. The presence of a stressful factor and a protective factor was evidenced in burnout: intrusive leadership and workaholism, respectively. Greater exhaustion was observed in workers belonging to generation X (41 to 55 years old). Mental fatigue is related to exhaustion in the productive, physical, and psychological dimensions of individuals. Addiction to work has moderated this phenomenon, however, it is urgent to limit and optimize work hours as well as promote disconnection and rest among workers within the framework of a healthy work policy.

## Abstract (pt)

Este estudo pretendeu analisar as evidências científicas disponíveis na literatura sobre a relação entre fadiga mental e esgotamento associados ao teletrabalho em tempos de pandemia de covid-19 mediante uma revisão integrativa. Esta revisão incluiu a pesquisa nos seguintes bancos de dados: PubMed, Scopus, Taylor & Francis, Embase, Science Direct e SciELO. Usando os descritores DeCs e MeSH de ciências da saúde, os artigos incluídos foram datados entre março e dezembro de 2021, durante a pandemia. De um total de 224 resultados, 215 artigos foram excluídos, e 9 foram considerados para a elaboração desta revisão integrativa. A fadiga mental foi relacionada ao tecnoestresse, à presença de sintomas somáticos, como ansiedade e insônia (p < 0,05) e à perda de motivação (p < 0,05). O esgotamento foi positivamente associada à sobrecarga, alta interdependência e falta de clareza quanto à função do trabalhador. Evidenciou-se a presença de um fator estressante e de um fator protetor em relação ao esgotamento, como a liderança intrusiva e o vício no trabalho, respectivamente. Observou-se maior exaustão nos trabalhadores da geração X (41 a 55 anos). Concluiu-se que a fadiga mental está relacionada à exaustão nas dimensões produtiva, física e psicológica dos sujeitos. A dependência do trabalho moderou esse fenômeno, porém, é urgente limitar e otimizar as horas de trabalho, bem como promover a desconexão e o descanso dos trabalhadores no âmbito de uma política de trabalho saudável.

## INTRODUCTION

During the COVID-19 pandemic, the online, telecommuting, and home-based work modalities were intensified for different types of work around the world in order to limit productive unemployment, adapt the operation of companies, and limit contagion. This was a complex health scenario with a very high demand for health care centers.^1^ Remote workers were approximately 40% in Canada,^2^ 71% in the United States,^3^ 60% in Nordic countries, and 37% in the European Union.^4^ Considering this work system, research has been emphatic in rescuing the positive aspects of this modality. Working from home provides greater flexibility, productivity, efficiency, and satisfaction.^5^ In this sense, this form of teleworking also saves commuting time and promotes faster work, mainly for workers who require greater concentration in solving complex problems, provided that the home office protects the worker from elements such as unexpected visits and constant distractions.^6^

On the other hand, and the issue addressed by this review, the telework system also brings forth negative elements, mainly in jobs that require interaction and collaboration with others. Executing tasks from one’s home becomes more difficult and slower due to social and professional isolation; the instances in which information is shared and cultivated are lacking. These links increase trust in work teams.^7^ The rapid adaptation to the online reality to which workers have been exposed has been compensated by excessive interaction and demand for time invested in connectivity,^8^ as well as a minimal separation between work and personal environments.^9^ This aspect is related to the constant disturbance and interruption of workspaces due to the need to also attend to domestic and family situations.^10^

Studies warn that stressors mentioned in remote jobs may be associated with a harmful overload reported by workers in different areas, characterized by the presence of fatigue, weariness, exhaustion, and somatization of signs and symptoms such as pain, increased anxiety, and sleep disorders.^11^

On the other hand, reports of negative elements associated with teleworking constitute an alert for creating new guidelines and redesigning this work modality to mitigate the associated risks and protect healthy working conditions that are in greater harmony with the human being.^12^ Recognizing the scope of these issues will allow us to address the most critical aspects that require adjustments to improve working conditions in the teleworking modality. The foregoing may play a moderating role in the regulatory frameworks designed for this work system, which may continue after the pandemic.

The purpose of this review is to explore the interactions between fatigue and burnout syndrome in teleworkers at different jobs and integrate the results found on this matter.

## METHODS

### STUDY DESIGN

This integrative review work was based on evidence of previous studies from the following stages: a) formulation of a research question; b) literature search; c) data collection; d) critical analysis of the included studies; e) summary of the main results; and f) presentation of the integrative review.

The following question was posed to guide our research: What is the relationship between mental fatigue and burnout syndrome due to home-based work or teleworking in different jobs during the pandemic?

The researchers used the PICO method (P: patient/problem; I: intervention; C: comparison; O: result/outcomes) to search for articles in the selected databases.

### STUDY IDENTIFICATION AND SELECTION

The search was carried out in the PubMed, Scopus, ScienceDirect, Taylor & Francis, Embase, and SciELO databases considering articles published between March and December 2021 in both Spanish and English. The search was performed using the Medical Subject Headings (MeSH) and Health Sciences (DeCs) thesaurus descriptors related to the objective of the review, linked with Boolean operators (AND) and (OR). The concepts included were “fatigue,” “mental,” “exhaustion,” “home,” “office,” “telework,” “remote work,” and “pandemic.”

Study selection was initially performed by reading the titles and abstracts. This phase also made it possible to eliminate articles that presented at least one exclusion criterion. In a second phase, the included articles were reviewed to ensure that they met all the inclusion criteria.

The inclusion criteria consisted of only original observational research papers and reviews that included the study of mental fatigue and burnout associated with teleworking during the pandemic, published between January and December 2021, available in English or Spanish.

The exclusion criteria consisted of studies that did not include at least three of the keywords in the title, case studies, book chapters, and conference papers. Articles published in years other than 2021 and in languages other than English and Spanish were also excluded.

Potentially relevant articles were reviewed by the researcher and an external researcher to determine their completeness. Discrepancies between the two researchers were resolved by a third author who acted as expert judgement.

### DATA EXTRACTION

The data extraction process was carried out by the researcher using a Microsoft Excel file detailing the author, year of publication, type of study and journal, design and sample size, prevalence of mental fatigue and burnout, and recommendations to the job. The methodological rigor of the studies was assessed using the Scottish Intercollegiate Guidelines Network (SIGN) Methodology Checklist tool. Only articles that reached the “high quality (++)” and “acceptable (+)” categories were included.

## RESULTS

**Figure 1:**
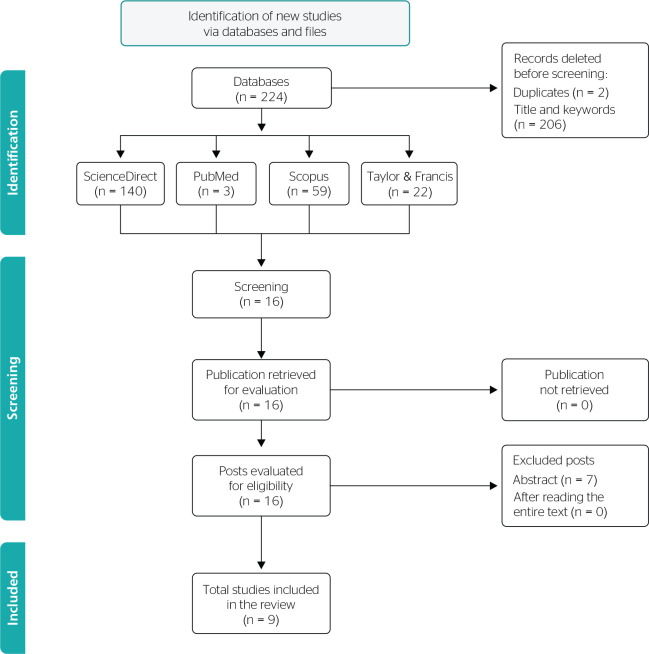
Methodology and criteria for study inclusion and selection.

After the search, 224 articles were found in the identification phase using the descriptor. In the selection stage, articles were excluded based on their unrelated titles and duplication. The eligibility criteria (by abstract and full text) led to nine articles that were finally included and analyzed (Figure 1).

The results of this analysis are shown in Table 1, which shows the main authors, titles, journals, objectives, main results, and future suggestions for the work according to the authors.

**Table 1:** Summary of the articles included in the review

Author	Title	Journal	Objective	Results	Work suggestions
Spieler & Baum^13^	Burnout: A mindful framework for the radiologist	Current Problems in Diagnostic Radiology	To identify the risk factors present in radiologists and promote prevention strategies and approaches.	The required darkness of 25-50 lux is related to decreased alertness and melatonin secretion. Excessive stationary and isolated work was shown to be related to musculoskeletal pain and depersonalization. Teleradiology has experienced over-demand as a medical support unit for services that do not have a face-to-face imaging area. Radiologists found it difficult to recognize exhaustion, with women being more susceptible. Greater burnout was observed in generation X (41-55 years old).	Promoting the use of online burnout self-assessment tools. Optimizing ergonomics by reducing mental load and minimizing distractions such as emails and calls, duration of breaks, and unifying the language for interpretations. Incorporating new workflows through interns for less complex tasks. Increasing resources for mental well-being and regular and systematic practice of mindfulness.
Ghasemi et al.^14^	Exploring unprecedented problems of academicians during the COVID-19 pandemic and their relationship with fatigue and mental health	Gene Reports	To explore the unprecedented problems of academicians during the COVID-19 pandemic and investigate the effects of these problems on their perceived fatigue and mental health.	Unprecedented problems can be categorized in two groups: family-related (parental burnout) and university-related (associated to technostress and difficulties with new technologies). Both dimensions of problems are related to increased mental fatigue, the presence of somatic symptoms (anxiety/insomnia) (p < 0.05), and reduced activity and motivation (p < 0.05). Both components were not significantly related to physical fatigue (p > 0.05).	Researchers indicate that it may be positive to include or explore other occupations of academicians in future studies. Authorities should take actions to remedy these problems, such as training academicians on the efficient use of new technologies for teaching and research activities.
Albro & McElfresh^15^	Job engagement and employeeorganization relationship among academic librarians in a modified work environment	The Journal of Academic Librarianship	To quantitatively examine job engagement and employee-organization relationship in librarians during the work-from-home period of the COVID-19 pandemic.	Job engagement and organizational commitment scores measured between April and September showed a decrease, being significantly different: [F (4,15) =7.355, p = 0.00174], and [t (26.334) = 2.3356, p = 0.02739], respectively, indicating a relationship between job engagement, employment duration, and organizational commitment scores, as well as a lower work engagement.	
Wütschert et al.^16^	Working from home: Cognitive irritation as a mediator of the link between perceived privacy and sleep problems.	Industrial Health	To investigate the impact of perceived privacy on cognitive irritation and sleep problems among employees who worked from home during the pandemic.	A lower privacy level perceived when working from home can cause cognitive irritation, a perceived lack of psychological detachment, and sleep problems (p < 0.05). The lack of privacy acts as a work stressor and could be related to mental and physical health issues.	Research suggests further studies on the effects and design of working from home. Future research is required on recovery strategies for teleworkers, mindfulness, and shorter breaks.
Barbieri et al.^17^	Don’t call it smart: working from home during the pandemic crisis	Frontiers in Psychology	To explore the demands-resources framework and whether and to what extent job demands, organizational job resources, and personal resources have affected the worker’s quality of life during the pandemic.	Isolation and workload exert a certain effect only through perceived stress (r = 0.17), while organizational support contributes to a better quality of life only by means of its influence on job satisfaction (r = 0.32). An increase in job demands has a negative impact on quality of life. Two mediator variables have relevant and opposite influence on quality of life, with a stronger negative effect of stress (r = -0.39) with respect to a positive impact of job satisfaction (r = 0.14). For women, having children younger than 18 years old has a positive effect on perceived stress (r = 0.10); having children in pre-scholar age significantly reduced reported job satisfaction (r = -0.11).	Acting to increase job satisfaction mainly in women working from home: autonomy, task variety and significance, skill variety and specialization, interdependence, social support, and feedback. Promoting and providing information technology support, timely information, and relevant work materials to cope with working conditions that are not always easy.
Mihalca et al.^18^	Exhaustion while teleworking during COVID-19: A moderated-mediation model of role clarity, self-efficacy, and task interdependence.	Oeconomia Copernicana	To investigate the link between work overload and employee well-being, considering role clarity as a mediator and emotional exhaustion moderated by task interdependence and self-efficacy in the job demands-resources model.	Work overload was positively and significantly associated with exhaustion (β = 0.82, p < 0.001) when controlling for negative emotionality (β = 0.64, p < 0.001). Role clarity was negatively related to emotional exhaustion (β = -0.27, p < 0.001). The interaction of role clarity and task interdependence has a significant effect on emotional exhaustion (β = - 0.24, = 0.09, p < 0.001). The highest level of exhaustion was predicted by work overload when task interdependence was high and role clarity was low. There is an interaction effect of role clarity and self-efficacy on teleworkers’ exhaustion (β = -0.21, = 0.10, p < 0.05), indicating that the link between role clarity and exhaustion was moderated by self-efficacy.	Organizations should aim at increasing teleworkers’ self-efficacy through leadership, feedback, or intensive trainings. Managers should identify employees who have high self-efficacy and/or those with low task interdependence, which are important in a context of high volumes of telework.
Martínez et al.^19^	Predictors of burnout in social workers: the COVID-19 pandemic as a scenario for analysis	International Journal of Environmental Research and Public Health	To determine the burnout levels in professions such as social work during the first wave of the pandemic.	High levels of emotional exhaustion (70.1%) and depersonalization (48.5%) were observed. Personal accomplishment was low (36.6%), and 70.8% of social workers believed they might need psychological or psychiatric treatment post COVID-19 pandemic; 79.5% of the participants said they did not feel recognized by the organization.	Emotional exhaustion in social workers can cause work-related psychosocial illnesses. Organizations should implement urgent measures to improve the working conditions of their professionals, as well as psychological and psychiatric care services for those most in need.
Magnavita et al.^20^	Telecommuting, time off work, and intrusive leadership in workers’ well-being	International Journal of Environmental Research and Public Health	To study the intrusive leadership style and the demand for time off work, which was associated with occupational stress, and to investigate if a workaholic attitude could increase the negative effects of intrusive leadership and the demand for time off work.	Intrusive leadership and working after hours were significantly associated with occupational stress. Furthermore, leadership style along with overtime work were associated with reduced happiness, anxiety, and depression. The overtime work behaves as a moderator between intrusive leadership, overtime, and job stress.	Organizations and companies should implement policies to prevent intrusive leadership and workaholism. In addition, they should guarantee the right to disconnect to decrease the effects of working outside traditional hours.
Bonanomi et al.^21^	Prevalence and health correlation to online fatigue: A cross-sectional study on the Italian academic community during the COVID-19 pandemic.	PLoS One	To validate an assessment tool (Online Fatigue Scale), estimate the prevalence of online fatigue in academic staff, and identify the correlations of Online Fatigue in terms of mental health and psychosomatic symptoms.	Two factors were identified: off-balance fatigue and virtual relations fatigue. In 45,3% of the participants, a significant association was observed between high levels of fatigue and technology use, while in 44,8% of individuals, a positive association was observed between high levels of fatigue and the use of platforms and videoconferences. Furthermore high levels of virtual relations fatigue were reported by 43.9% and 45.1% of participants who lived alone or were not cohabiting, respectively (difference not significant). High and medium levels of both factors were associated with a higher frequency of symptoms such as muscle tension, irritability, visual disturbances, fatigue, palpitations, and mood alterations. Both dimensions did not vary across different age, academic role, or gender profiles.	University administrators should implement effective interventions to relieve the mental burden and reduce the fatigue level and contribute to the construction of safe and healthy work environments.

## DISCUSSION

After analyzing the articles included in this review, risk factors and stressors associated with teleworking become evident. Nevertheless, protective and moderating factors of work overload in a virtual environment were also observed.

The first stressor corresponds to what is evidenced in the work of Spieler & Baum,^13^ who warn that the overexposure to computer screens for much of the day has been related to tiring effects on attention, concentration, and visual fatigue for different groups, ages, and genders.^22^ On the other hand, static postural overload was related to body pain and muscle tension.^23,24^

Another criterion includes technostress and technological difficulties linked to connection problems, platform management, and incompetence to deal with Information and Communications Technology (ICT), found by Bonanomi et al.^21^ and Ghasemi et al.^14^ This has been related to a greater demand for time invested in solving technical problems, which delays the progress of work tasks and was linked to greater anxiety and cognitive irritation.^25-27^

Regarding the work environment of Martínez et al.^19^ and Mihalca et al.,^18^ intrusive leadership and over-hours contributed negatively to worker performance and led to stress, insomnia, depression signs and symptoms, loss of motivation, mental exhaustion, depersonalization, and increased need for psychological or psychiatric care.^28^

Although teleworking has had negative effects, aspects that would act as protectors against overload have been described, mitigating and buffering the mental workload; these include workaholism and recognition of the company towards its workers,^29^ being in line with the results found by Magnavita et al.^20^

Additionally, studies by Barbieri et al.^17^ reported that women had lower self-efficacy and job satisfaction when having to solve domestic problems and caring for preschool children. In this sense, women have experienced the overload of reconciling work, domestic, family, and parenting activities.^30^

To mitigate and counteract the effects described as detrimental to occupational health, various initiatives derived from studies and research aim to make adjustments that address both work systems and good employer practices.^31,32^

Among the suggestions proposed by the authors,^13,14,17,18^ those focused on work systems aim to: a) manage workers’ self-assessment early, allowing them to recognize exhaustion and intervene promptly; b) limit distractions and shorten work times by improving efficiency; c) provide brief training in the use of technology and simultaneous support for problem-solving; d) improve the identification of workers’ skills to redesign roles, tasks, and promote autonomy.

On the other hand, proposals related to the companies’ good practices^16,20,21^ suggest: a) increasing the duration of rest times associated with the systematic guided practice of mindfulness or meditation; b) providing the assistance of professional psychologists for their workers; c) preventing work addiction and promoting disconnection through free time.

Recent studies suggest reviewing the impact that the creation of new telecommuting jobs could have and improving the separation between work and non-work domains to mitigate the negative effects related to exhaustion, mental fatigue, and tiredness by overload.^33^

Another important recommendation is to review the correlation between the frequency and duration of calls and videoconferences per working day with the mental processes that mediate these activities such as attention and concentration, as well as production processes through performance.^34^ The foregoing will make it possible to estimate whether reducing the number of calls and/or online time during teleworking is feasible.

### STUDY LIMITATIONS

The limitations of this work are mainly the heterogeneity of the primary studies and those included in the review. Second, the lack of new studies from the citation and/or organization search, which is why the final number of included studies was low.

## CONCLUSIONS

Although working from home rescues positive aspects in times of a pandemic, this work allows us to visualize and conclude that the organizational conditions of work have deteriorated and there is real exposure to stressors that harm the mental, productive, and physical dimensions of individuals. It is necessary to review the maturity and critical awareness of the exposure of workers to the immediacy and to examine suggestive surveillance tools such as telepressure via control devices in contexts of overload.

In this sense, we should reflect and promote research that includes a greater homogenization of studies to allow better comparisons, evaluate the differences between men and women who, as evidenced, assimilate stressors differently and have different moderating and/or protective mechanisms.

Along this line, incorporating works that evaluate preand post-intervention measures could also allow discussing the efficiency of resources and strategies used to reduce the negative effects of teleworking.
